# Tuning curves vs. population responses, and perceptual consequences of receptive-field remapping

**DOI:** 10.3389/fncom.2022.1060757

**Published:** 2023-01-13

**Authors:** Ning Qian, Michael E. Goldberg, Mingsha Zhang

**Affiliations:** ^1^Department of Neuroscience and Zuckerman Institute, Columbia University, New York, NY, United States; ^2^Department of Physiology and Cellular Biophysics, Columbia University, New York, NY, United States; ^3^Departments of Neurology, Psychiatry, and Ophthalmology, Columbia University, New York, NY, United States; ^4^State Key Laboratory of Cognitive Neuroscience and Learning, IDG/McGovern Institute for Brain Research, Beijing Normal University, Beijing, China

**Keywords:** predictive remapping, forward expansion, LIP, FEF, transsaccadic visual stability, corollary discharge, space perception

## Abstract

Sensory processing is often studied by examining how a given neuron responds to a parameterized set of stimuli (tuning curve) or how a given stimulus evokes responses from a parameterized set of neurons (population response). Although tuning curves and the corresponding population responses contain the same information, they can have different properties. These differences are known to be important because the perception of a stimulus should be decoded from its population response, not from any single tuning curve. The differences are less studied in the spatial domain where a cell's spatial tuning curve is simply its receptive field (RF) profile. Here, we focus on evaluating the common belief that perrisaccadic forward and convergent RF shifts lead to forward (translational) and convergent (compressive) perceptual mislocalization, respectively, and investigate the effects of three related factors: decoders' awareness of RF shifts, changes of cells' covering density near attentional locus (the saccade target), and attentional response modulation. We find that RF shifts *alone* produce either no shift or an opposite shift of the population responses depending on whether or not decoders are aware of the RF shifts. Thus, forward RF shifts do not predict forward mislocalization. However, convergent RF shifts change cells' covering density for aware decoders (but not for unaware decoders) which may predict convergent mislocalization. Finally, attentional modulation adds a convergent component to population responses for stimuli near the target. We simulate the combined effects of these factors and discuss the results with extant mislocalization data. We speculate that perisaccadic mislocalization might be the flash-lag effect unrelated to perisaccadic RF remapping but to resolve the issue, one has to address the question of whether or not perceptual decoders are aware of RF shifts.

## Introduction

Tuning curves and population responses are among the most useful concepts in sensory studies. Consider, for example, a neuron with preferred orientation *x*_*p*_, and write its response to stimulus orientation *x*_*s*_ as *f* (*x*_*p*_, *x*_*s*_). If we fix the preferred orientation *x*_*p*_ of a neuron and plot response *f* as a function of a range of stimulus orientations *x*_*s*_, we obtain a tuning curve. On the other hand, if we fix the stimulus orientation *x*_*s*_ and plot *f* as a function of a set of cells' preferred orientations *x*_*p*_, we obtain a population response. Thus, a collection of tuning curves of different cells and the corresponding collection of population responses for different stimuli contain the same information; they just slice the same function *f* (*x*_*p*_, *x*_*s*_) along the different axes of the independent variables.

Despite their close relationship, tuning curves and population responses can be different in important ways. For example, it is known that when tuning curves shift in one direction, the corresponding population responses shift in the opposite direction (Gilbert and Wiesel, 1990; Suzuki and Cavanagh, 1997; Yao and Dan, 2001; Teich and Qian, 2003, 2010) (This is under the assumption that the decoders are unaware of the tuning shifts, a point we will elaborate below.). In the domain of stereovision, binocular phase-shifts and position-shifts between cells' RFs in the two eyes produce similarly unreliable disparity tuning curves, but the former generate more reliable population responses than do the latter (Chen and Qian, 2004; Tsang and Shi, 2004; Li and Qian, 2015). The differences between tuning curves and population responses are particularly important when perception is studied. Our perception of a stimulus must depend on relevant cells' population responses to that stimulus, instead on any single cell's responses to different stimuli (tuning curve). If a condition or manipulation changes population responses and tuning curves differently, then one must use population responses, not tuning curves, to predict the perceptual consequences.

**Figure 1:**
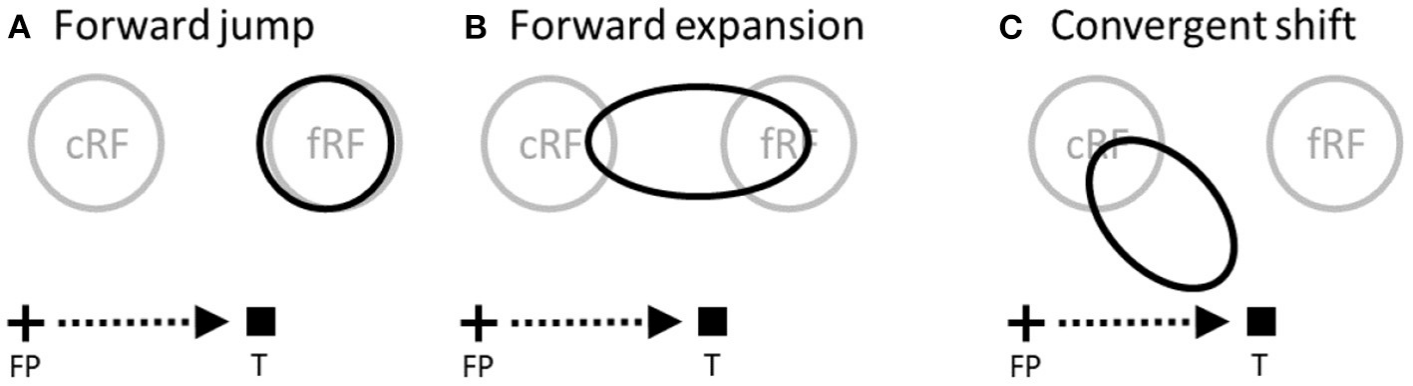
Perisaccadic RF remapping, drawn on the display screen for the stimuli. The cross, square and arrow represent the fixation point (FP), saccade target (T), and saccade vector, respectively. cRF and fRF refer to a cell's current (pre-saccadic) and future (post-saccadic) RFs, respectively. In each panel, the region(s) enclosed by black curve(s) represent perisaccadic RF (pRF). **(A)** Forward jump to fRF. **(B)** Forward expansion toward fRF. Both **(A)** and **(B)** will be referred to as forward shift. **(C)** Convergent shift toward the target.

In the spatial domain, cells' spatial tuning curves are simply their RF profiles. Around saccade onset, two types of RF changes, known as forward and convergent remapping, have been found in lateral intraparietal area (LIP), frontal eye fields (FEF), and other brain areas (Duhamel et al., 1992; Umeno and Goldberg, 1997; Kusunoki and Goldberg, 2003; Zirnsak et al., 2014; Neupane et al., 2016; Wang et al., 2016). Forward remapping is the shift of a cell's perisaccadic RF (pRF) from current (pre-saccadic) RF (cRF) toward its future (post-saccadic) RF (fRF) in the direction of the pending saccade (Figures 1A, B) whereas convergent remapping is the pRF shift toward the saccade target (Figure 1C). Further studies suggest that the two types of remapping originate from corollary discharge (CD) of saccade commands and attention at the target, respectively (Sommer and Wurtz, 2006; Neupane et al., 2016; Yang et al., 2019). Here we focus on the perceptual consequences, instead of the origins, of the remapping. There are also two types of perisaccadic perceptual mislocalization reported in the literature: forward (translational) in the direction of the pending saccade and convergent (compressive) toward the saccade target (Matin and Pearce, 1965; Honda, 1991; Ross et al., 1997; Lappe et al., 2000; Schlag and Schlag-Rey, 2002). Given such apparent correspondence between physiology and perception, it is often assumed that the two types of RF remapping generate the two types of perceptual mislocalization, respectively (Ross et al., 2001; Zirnsak et al., 2014). We will call this the *same-direction assumption* as it posits that RF shifts in a direction produce perceptual mislocalization in the same direction. In this paper, we evaluate this assumption in great detail. The above-mentioned differences between tuning curves and population responses should already cast some doubts on the assumption, but as we will see, the problem is further complicated by other factors including decoders' awareness of RF shifts, the RF-convergence induced change of cells' covering density for aware decoders, and attentional modulation of responses around the saccade target.

## Results

**Figure 2:**
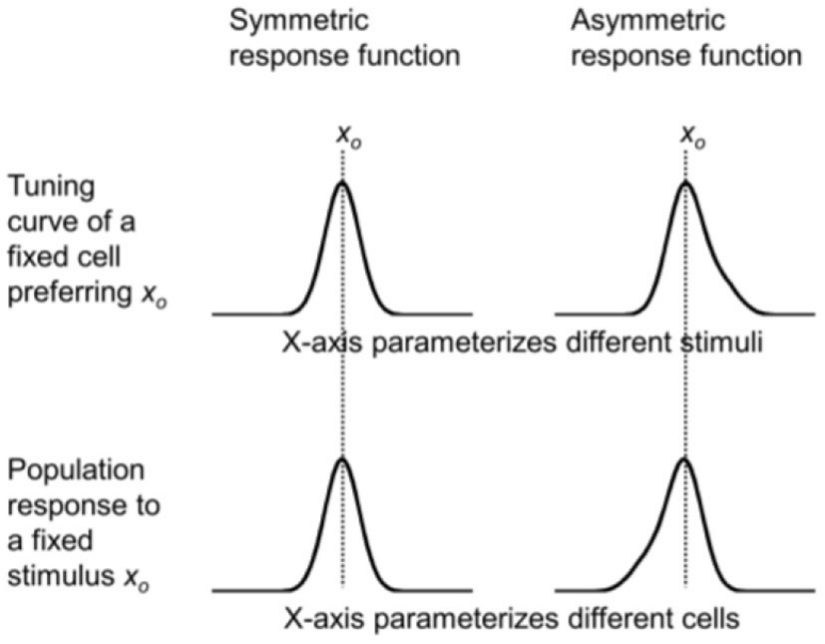
Simulations of the mirror relationship between the tuning curve of a cell preferring *x*_*p*_ = *x*_*o*_ **(top row)** and the population response to a stimulus *x*_*s*_ = *x*_*o*_ **(bottom row)**, under the assumption of translational invariance. The mirror relationship is hidden when a symmetric response function is used **(left column)**, but is revealed with an asymmetric function **(right column)**.

We first examine the relationship between tuning curves and population responses in a simple case to develop intuition. If a stimulus attribute (orientation, direction, spatial location, etc.) can be parameterized by *x*, then let *f* (*x*_*p*_, *x*_*s*_) represent how much a cell preferring stimulus *x*_*p*_ responds to input stimulus *x*_*s*_. In the case of spatial RFs, *x*_*p*_ and *x*_*s*_ are two-dimensional (2D) vectors representing the preferred position and stimulus position on the retina, respectively. The following discussion holds regardless of whether *x* is a scalar or 2D vector. For simplicity, simulations in this paper consider only one spatial dimension. Assume


(1) $$f(x_{p},\;x_{s})\;=\;f(x_{p}\;-\;x_{s})$$

namely that the response depends only on the difference between *x*_*p*_ and *x*_*s*_. This is the commonly assumed translational invariance. It is a good approximation if we view *f* (*x*_*p*_, *x*_*s*_) as representing the average response of all cells with the same *x*_*p*_, and the parameter range is limited so that, for example, we do not need to consider the difference between fovea and periphery (We will relax this assumption later.). Then the tuning curve of a cell preferring *x*_*p*_ = *x*_*o*_ [i.e., *f* (*x*_*o*_ – *x*_*s*_) as a function of *x*_*s*_] and the population response to a stimulus *x*_*s*_= *x*_*o*_ [i.e., *f* (*x*_*p*_ – *x*_*o*_) as a function of *x*_*p*_] are exact mirror images of each other with respect to *x*_*o*_. If *f* (*x*_*p*_ – *x*_*s*_) is even symmetric with respect to *x*_*p*_ = *x*_*s*_ (as is often the case for some commonly used functions such as Gaussian), then the tuning curve of a cell preferring *x*_*o*_ and the population response to stimulus *x*_*o*_ are the same (Figure 2, left column). Perhaps for this reason, tuning curves and population responses are often viewed as the same. However, their mirror relationship becomes obvious with an asymmetric response function (Figure 2, right column).

**Figure 3:**
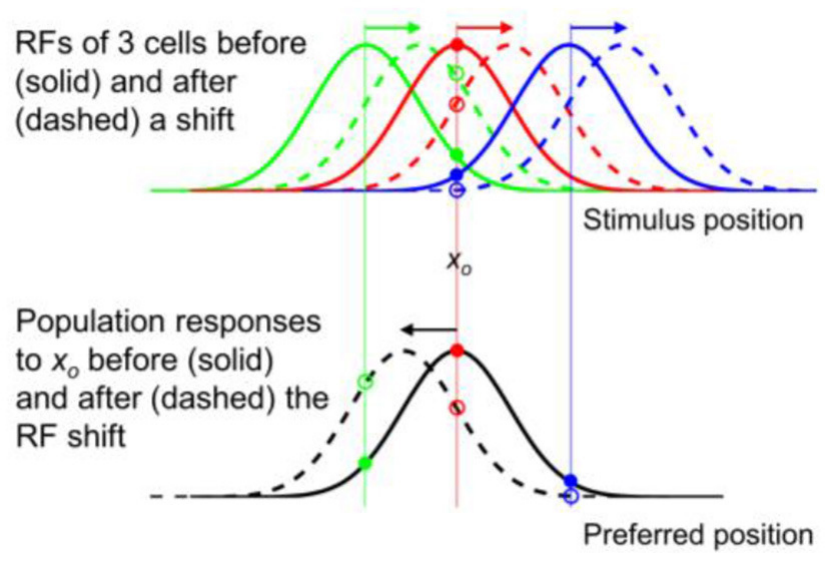
Opposite shifts of tuning curves (here RFs) and the population response for unaware decoders (after figure 6 of Yao and Dan, 2001). **(Top)** RFs of three arbitrary cells before (solid) and after (dashed) a rightward translation (rightward arrows), similar to the forward pRF jump in Figure 1A. *x*_*o*_ indicates a specific stimulus position which evokes responses from the cells before (filled dots) and after (open dots) the shift. **(Bottom)** The population responses of all cells to *x*_*o*_ before (solid black) and after (dashed black) the RF shifts, plotted here at the cells' pre-shift preferred positions (unaware decoders). The three cells' responses from the top panel are indicated. If the cells' post-shift responses are plotted at their post-shift preferred positions (aware decoders), then the pre- and post-shift population responses are identical (both solid black).

Now consider the situation where all the tuning curves translate by an amount *d*. This means that the independent variable *x*_*s*_ in the tuning function *f* (*x*_*p*_ – *x*_*s*_) should be replaced by (*x*_*s*_ – *d*) to produce the new function *f* [*x*_*p*_ – (*x*_*s*_– *d*)]. However, since


(2) $$f\left(x_{p}\;-\;(x_{s}\;-\;d)\right)\;=\;f\left((x_{p}\;+\;d)\;-\;x_{s}\right)$$

shifting tuning curves (as a function of *x*_*s*_) by *d* is equivalent to shifting the corresponding population response (as a function of *x*_*p*_) by negative *d*. This is an algebraic demonstration of the known result that when tuning curves shift in one direction, the corresponding population responses shift in the opposite direction (Gilbert and Wiesel, 1990; Suzuki and Cavanagh, 1997; Yao and Dan, 2001; Teich and Qian, 2003, 2010). A related algebraic demonstration appeared in the appendix of Teich and Qian (2010). The opposite shifts between tuning curves and population responses are a consequence of their mirror relationship, and can be seen regardless of whether the response function *f* (*x*_*p*_ – *x*_*s*_) is symmetric or not; the simulations in Figure 3 use a symmetric (Gaussian) function.

There is an implicit assumption in the above demonstration, namely that the decoder is unaware of the tuning shift so that the post-shift population response is plotted against the cells' *pre-shift* preferred parameter *x*_*p*_. If, instead, the decoder *is* aware of the tuning shift so that the post-shift population response is plotted against the cells' *post-shift* preferred parameter ${x^{\prime }}_{p}\;=\;x_{p}\;+\;d$, then Eq. 2 becomes:


(3) $$f\left((x_{p}+d)\;-\;x_{s}\right)\;=\;f({x^{\prime }}_{p}\;-\;x_{s})$$

Therefore, the post-shift population response $f({x^{\prime }}_{p}-x_{s})$ as a function of post-shift preferred parameter ${x^{\prime }}_{p}$ is identical to the pre-shift population response *f* (*x*_*p*_ – *x*_*s*_) as a function of pre-shift preferred parameter *x*_*p*_. That is, for decoders aware of the tuning shift, the corresponding population responses, and hence perception, do not shift. [See Teich and Qian (2003) for related work on plotting post-adaptation population response as a function of pre- and post-adaptation preferred orientations.]

These results are simulated in Figure 3 for spatial tuning (RFs). The top panel shows RFs of three example cells (colored green, red, and blue) before (solid) and after (dashed) a rightward shift. The bottom panel shows the population response of all cells to stimulus position *x*_*o*_ before (solid black) and after (dashed black) the RF shift, as a function of the pre-shift preferred positions (unaware decoders). Note the mirror relationship between the dashed red curve and the dashed black curve with respect to *x*_*o*_. If the cells' post-shift responses are plotted at their post-shift preferred positions (aware decoders), then the pre- and post-shift population responses will be identical (both solid black). Thus, for a tuning (RF) translation, the aware and unaware decoders should report no mislocalization and an opposite mislocalization, respectively, contradicting the same-direction assumption.

The above conclusion can be understood intuitively. Consider, for example, the “red” cell in the top panel of Figure 3 whose pre- and post-shift RFs are represented by the solid and dashed red curves, respectively. If the decoder is “unaware” of the shift, then whenever the cell fires, it is evidence that a stimulus appears at the peak position of the solid red curve. Now after the RF shift, the cell fires maximally to a stimulus at the peak position of the dashed red curve but the decoder will still view that as strong evidence for a stimulus at the peak position of the solid red curve. Thus a rightward RF shift contributes to a leftward shift of the decoded position. If, on the other hand, the decoder is “aware” of the RF shift, then a cell's firing is evidence for stimulation at the peak position of its current RF. So after the RF shift, the decoder will view the red cell's maximal firing to a stimulus at the peak position of the dashed red curve as strong evidence for a stimulus at the same position, and thus no perceptual mislocalization.

With the above basic understanding of the relationship between tuning curves and population responses, we now turn to the perceptual consequences of various types RF remapping. The simplest type is perisaccadic forward jump (Duhamel et al., 1992; Kusunoki and Goldberg, 2003): around saccade onset, cells respond to stimuli in their post-saccadic RFs (future RFs or fRFs) with a concurrent reduction of their responses to stimuli in their current RFs (cRFs). This can be approximated as a translation of perisaccadic RFs (pRFs) in the saccade direction by the amount of the saccade amplitude (Figure 1A). Therefore, the above analysis of tuning shift applies, with *d* equal to the saccade amplitude. We conclude that population responses (and thus perception) should show either no shift or a backward shift against the saccade direction depending on whether or not decoders are aware of the forward RF jump. The conclusion is the same even if the RF shift *d* is not equal to the saccade amplitude.

Next we consider perisaccadic forward RF expansion (Wang et al., 2016): a closer examination shows that LIP perisaccadic remapping is a progressive shift of a cell's cRF toward its fRF over several tens of msec. When the shifting pRF is integrated over this time window, it appears as a forward expansion covering the region between the cRF and fRF (Figure 1B). If perceptual decoders are fast enough to resolve pRFs' progressive shift over time, then the conclusion is basically a time-varying version of Figure 3: the population responses (and thus perception) should show either no shift or a progressive backward shift against the saccade direction depending on whether or not decoders are aware of the RF progression.

**Figure 4:**
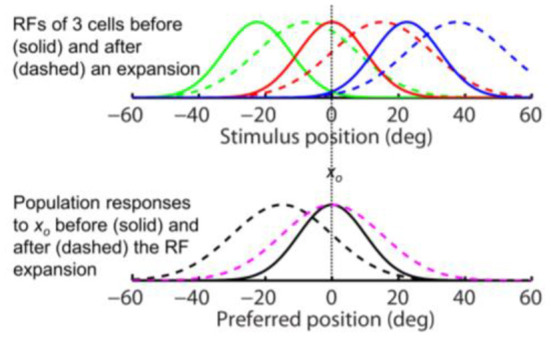
The same-direction assumption is false for forward RF expansion as is for forward RF jump in Figure 3. **(Top)** RFs of three arbitrary cells before (solid) and after (dashed) a perisaccadic rightward expansion. **(Bottom)** The population responses of all cells to *x*_*o*_ = 0 before (solid) and after (dashed) the RF expansion. The dashed black and magenta curves are the post-expansion responses plotted against the pre- and post-expansion preferred positions, for the unaware and aware decoders, respectively.

If, on the other hand, perceptual decoders operate at a time scale significantly longer than that of pRF progression, then they effectively integrate a cell's shifting pRF as a spatial expansion, with a center between the cRF and fRF (Figure 1B). In this case, we can express the pRFs by replacing the tuning function *f* (*x*_*p*_ – *x*_*s*_) by *f* ([*x*_*p*_ – (*x*_*s*_– *d*)]/*k*) where center shift *d* is less than the saccade amplitude and *k* > 1 is the RF expansion factor. Since, similar to Eq. 2, we have:


(4) $$f\left([x_{p}\;-(x_{s}\;-d)]/k\right)\;=\;f\left([(x_{p}\;+\;d)\;-x_{s}]/k\right)$$

the above conclusions for the forward RF jump hold for the forward RF expansion, the only difference being that the RF expansion factor *k* increases the width of the population responses for both the aware (dashed magenta) and unaware (dashed black) decoders in the simulations of Figure 4, as expected from Eq. 4. For aware decoders, the RF expansion does not change the peak location of the population responses (cf. solid black and dashed magenta). We conclude that for both types of forward RF shifts, the population response (and thus perception) shows either no shift or a backward shift, depending on whether or not decoders are aware of the RF shifts.

We can remove the translational-invariance assumption used above by considering the dependence of RF size on eccentricity. So instead of Eq. 1, we now assume:


(5) $$f\left(x_{p},\;x_{s}\right)=\;f\left(\left[x_{p}\;-\;x_{s}]/[a\left|x_{p}\right|+1\right]\right)$$

where position vectors *x*'s are all measured from the fovea as the origin, and *a* > 0 is a constant. [*a* = 0 would reduce Eq. 5 to Eq. 1.] To understand Eq. 5, first note that for a cell with preferred position vector *x*_*p*_ from the fovea, its eccentricity is given by the norm |*x*_*p*_|. Thus, the factor (*a*|*x*_*p*_| + 1) ≥ 1 simply scales up the RF size with its distance |*x*_*p*_| from the fovea, and *f* without the scaling (the factor equals 1) determines the RF size at the fovea where |*x*_*p*_| = 0.

**Figure 5:**
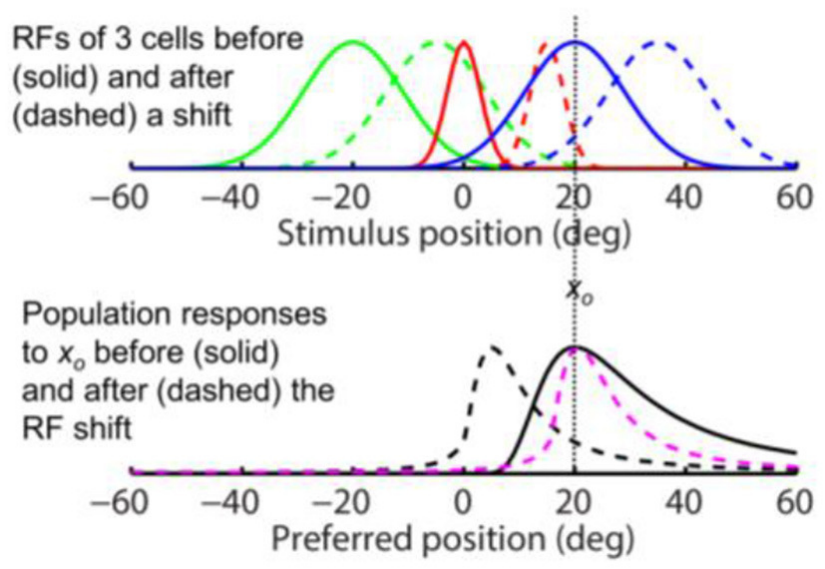
Removing translational invariance does not change the conclusion that the same-direction assumption is false for forward RF shifts. This figure assumes that a cell's shifted RF size is determined by its *pre*-shift eccentricity. The *x*-axes measure position as eccentricity from fovea (0). **(Top)** RFs of three arbitrary cells before (solid) and after (dashed) a perisaccadic rightward jump. **(Bottom)** the population responses of all cells to a stimulus at *x*_*o*_ = 20 deg before (solid) and after (dashed) the RF shift. The dashed black and magenta curves are the post-shift responses plotted against the pre- and post-shift preferred positions, for the unaware and aware decoders, respectively.

**Figure 6:**
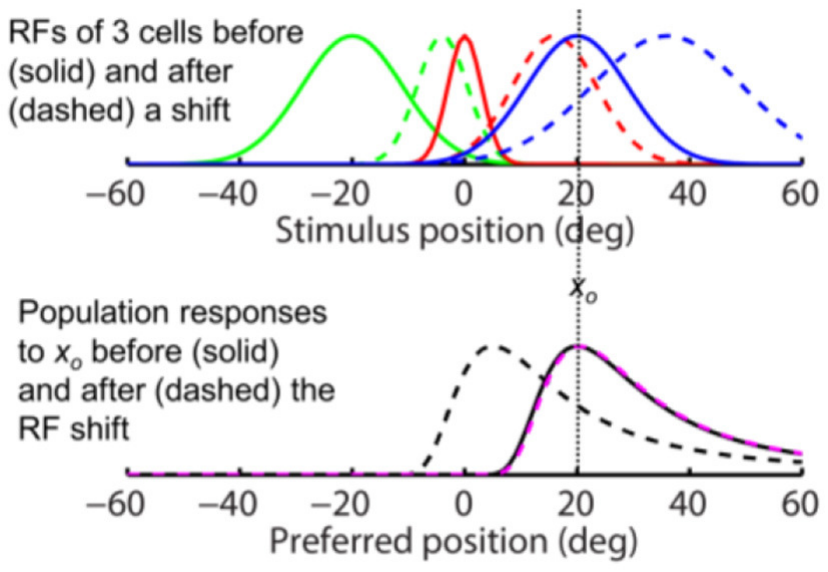
Removing translational invariance does not change the conclusion that the same-direction assumption is false for forward RF shifts. This figure assumes that a cell's shifted RF size is determined by its *post*-shift eccentricity. The format is identical to that for Figure 5.

Now consider the forward RF jump in the context of eccentricity dependence of RF size (the forward expansion case can be similarly treated). When a cell's RF shifts to a new position by *d*, its RF size may be determined by either the pre-shift eccentricity |*x*_*p*_| or the post-shift eccentricity |*x*_*p*_+*d*|. Since we do not know which case is true, we consider both. We can represent the post-shift response function as *f* ([*x*_*p*_– (*x*_*s*_– *d*)]/[*a*|*x*_*p*_| + 1]) and *f* ([*x*_*p*_– (*x*_*s*_– *d*)]/[*a*|*x*_*p*_+ *d*| + 1]), for the two cases respectively. Since we have


(6) $$\begin{array}{l} \;f\left(\left[x_{p}-(x_{s}-d)]/[a\left|x_{p}\right|+1\right]\right)=\\ f([(x_{p}+d)\;-\;x_{s}]/[a|x_{p}|+1]) \end{array}$$

and


(7) $$\begin{array}{l} \;f\left(\left[x_{p}-(x_{s}-d)]/\;[a\left|x_{p}+d\right|+1\right]\right)=\\ f([(x_{p}+d)-\;x_{s}]/[a\left|x_{p}+d\right|+\;1]) \end{array}$$

for the two cases, the above conclusions on the differences between RF shifts and population-response shifts remain valid. This is confirmed by simulations in Figures 5, 6 for the two cases, respectively. In the top panel of Figure 5, since a cell's shifted RF size is determined by its *pre*-shift eccentricity, its size does not change with the shift (the dashed and solid RF curves of the same color have the same width). In the top panel of Figure 6, in contrast, a cell's shifted RF size is determined by its *post*-shift eccentricity. For example, the “green” cell shifts to a smaller eccentricity and thus has a smaller post-shift size (the dashed green curve has a smaller width than the solid green curve). In both cases, the RF shifts produce either no shift or an opposite shift of the population response, depending on whether or not decoders are aware of the RF shifts. Thus, removing translational invariance does not change the conclusion that the same direction assumption is false.

Note that in Figures 5, 6, even though the RFs are symmetric (with respect to the preferred positions), the population responses can be asymmetric because of the eccentricity dependence of the RF sizes. Also note that for decoders aware of the RF shift, the pre- and post-shift population responses are identical in Figure 6 (solid black and dashed magenta) but not in Figure 5. This can be understood *via* the corresponding Eqs 7 and 6. When Eq. 7 is plotted as a function of the post-shift preferred position $x_{p}^{\prime }\;=\;x_{p}+d$, it is identical to the pre-shift response *f* ([*x*_*p*_ – *x*_*s*_]/[*a*|*x*_*p*_| + 1]) as a function of the pre-shift preferred position *x*_*p*_. This is not true for Eq. 6.

**Figure 7:**
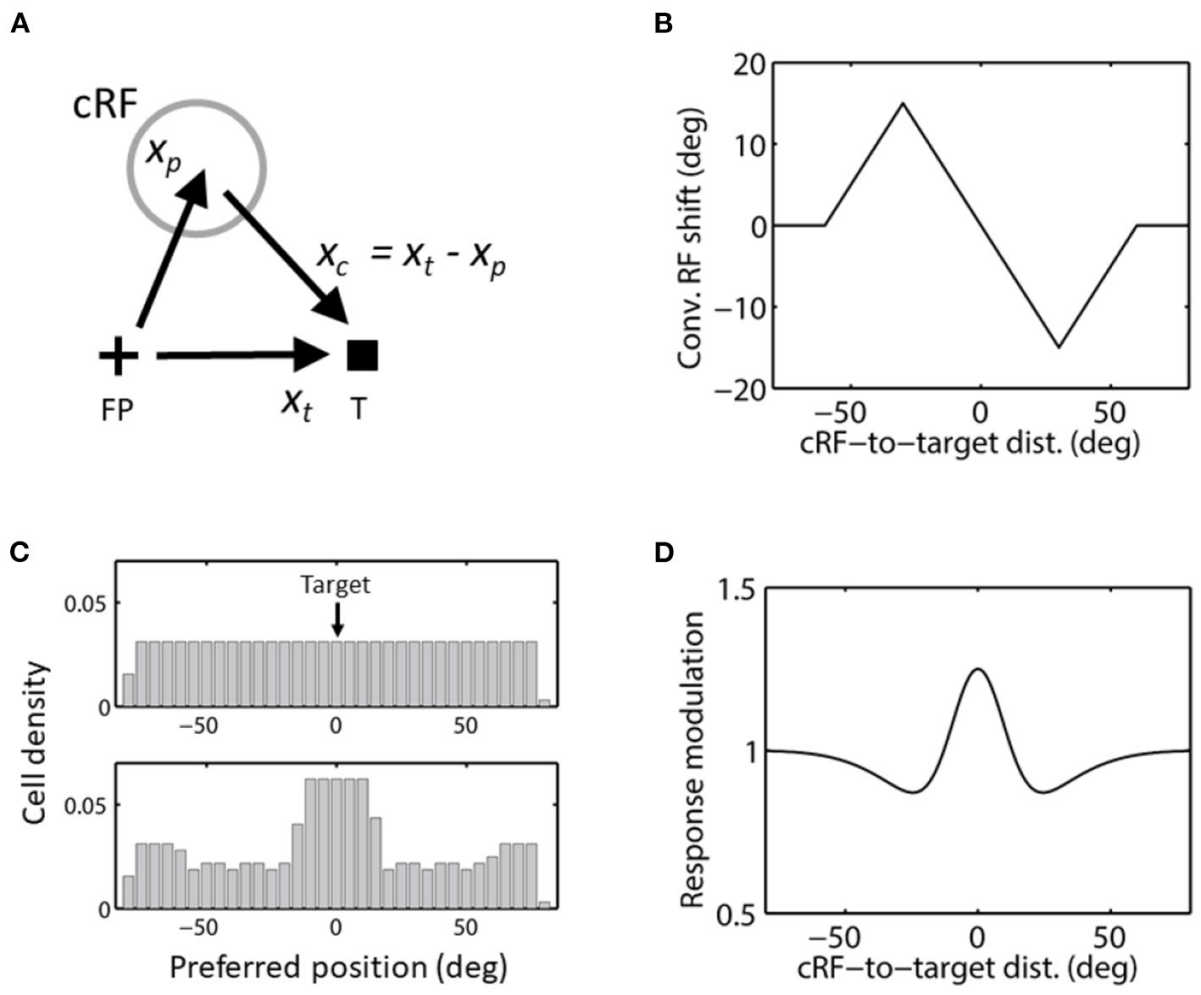
Convergent RF shift. **(A)** The direction of convergent RF shift. FP and T indicate the initial fixation point and saccade target, respectively. **(B)** Convergent RF shift as a function of the cRF-to-target distance used in the simulations of Figures 8–10. cRFs on the left and right side of the target (at 0) shift to the right (positive) and left (negative), respectively, as they converge to the target. **(C)** The cells' covering density before (top) and after (bottom) the convergent RF shifts toward the target in c, for aware decoders. The density stays the same (top) for unaware decoders. **(D)** The center/surround attentional modulation as a function of the cRF-to-target distance, used in the simulations of Figure 9. This curve is scaled by a factor of 4 in the simulations of Figure 10.

We finally consider convergent RF remapping (Figure 1C). Let *x*_*t*_ represent the target position vector, then for a cell whose cRF prefers position vector *x*_*p*_, the convergent shift is in the direction of the vector (Figure 7A):


(8) $$x_{c}=x_{t}\;-\;x_{p}$$

Since the convergence is likely due to the attention at the target (Connor et al., 1996; Zirnsak et al., 2014; Neupane et al., 2016; Wang et al., 2019; Yang et al., 2019), the magnitude of the convergence must depend on the cRF-to-target distance |*x*_*c*_|. Thus the convergence shift vector can be represented by c(|*x*_*c*_|) *x*_*c*_, where the function c(|*x*_*c*_|) satisfies 0 ≤ c(|*x*_*c*_|) ≤ 1 to ensure that the shift is between the cRF and target. For simplicity, we assume translational invariance (Eq. 1) before the shift. Then the response function after the shift can be obtained by replacing *f* (*x*_*p*_ – *x*_*s*_) by *f* (*x*_*p*_ – [*x*_*s*_– c(|*x*_*c*_|) *x*_*c*_]). Since


(9) $$f(x_{p}\;-\;[x_{s}\;-\;c(|x_{c}|)\;x_{c}])=f([x_{p}+c(|x_{c}|)x_{c}\;]\;-\;x_{s})$$

we see once again the familiar pattern: for decoders unaware of the RF convergence, the population response shifts in the opposite, divergent directions, away from the target. If, on the other hand, decoders are aware of the RF convergence, the population response plotted as a function of the new preferred position $x_{p}^{\prime }\;=\;x_{p}+c\left(\left|x_{c}\right|\right)x_{c}$ is $f\left(x_{p}^{\prime }\;-\;x_{s}\right)$, identical to the pre-shift population response *f* (*x*_*p*_ – *x*_*s*_) as a function of the pre-shift preferred position *x*_*p*_.

Before we draw conclusions on perceptual consequences of convergent RF shifts, we need to consider a new factor: for aware decoders, convergent RF shifts change the density distribution of cells covering different positions. First note that this is not an issue for unware decoders which, by definition, do not “know” the RF shifts and always attribute a cell's response to its original (pre-shift) preferred position. As such, from the perspective of unware decoders, there is no change of cells' preferred positions and thus no change of the cells' covering density. In contrast, aware decoders attribute a cell's response to its new (post-shift) preferred position, and from their perspective, convergent RF shifts toward the target must change the cells' covering density. Also note that in the above discussions of the *forward* RF shifts, a uniform translation of RFs (with or without an expansion) in the saccade direction does not change the cells' covering density (Obviously, if future experiments find a non-uniform forward-shift pattern across space, then we will have to consider the change of cells' covering density for aware decoders.).

How a change of cells' covering density affects perceptual decoding depends on whether or not a given decoder uses the covering density. We showed above that for aware decoders, convergent RF shifts do not change the functional form of population responses. If a *specific* aware decoder uses the center-of-mass (mean) of a population response to represent perception, then the increased cell density tuned to the target must bias perception toward the target. In other words, even without any change to the shape of the population response, a decoder that take into account the changed sampling from the population response will generate a convergent (compressive) mislocalization toward the target. In contrast, if we use another specific aware decoder that identifies the peak (mode) of a population response as perception, then the cell-density change does not matter and convergent RF shifts do not produce perceptual mislocalization.

We ran simulations using a size of convergent RF shifts that first increases linearly with the cRF-to-target distance up to 30 deg and then decreases linearly to 0 up to 60 deg (Figure 7B). This is based on a circuit model for convergence RF shifts (Wang et al., 2019), and is consistent with the available data (Zirnsak et al., 2014; Yang et al., 2019). Intuitively, convergent RF shift size must be small at both small and large cRF-to-target distances, with a maximum at an intermediate distance: when the distance is small, there is not much room for the cRF to shift to the target and when the distance is large, the attentional effect at the cRF is diminished. For aware decoders, we show the cell densities covering different locations before and after the convergent shifts in Figure 7C.

**Figure 8:**
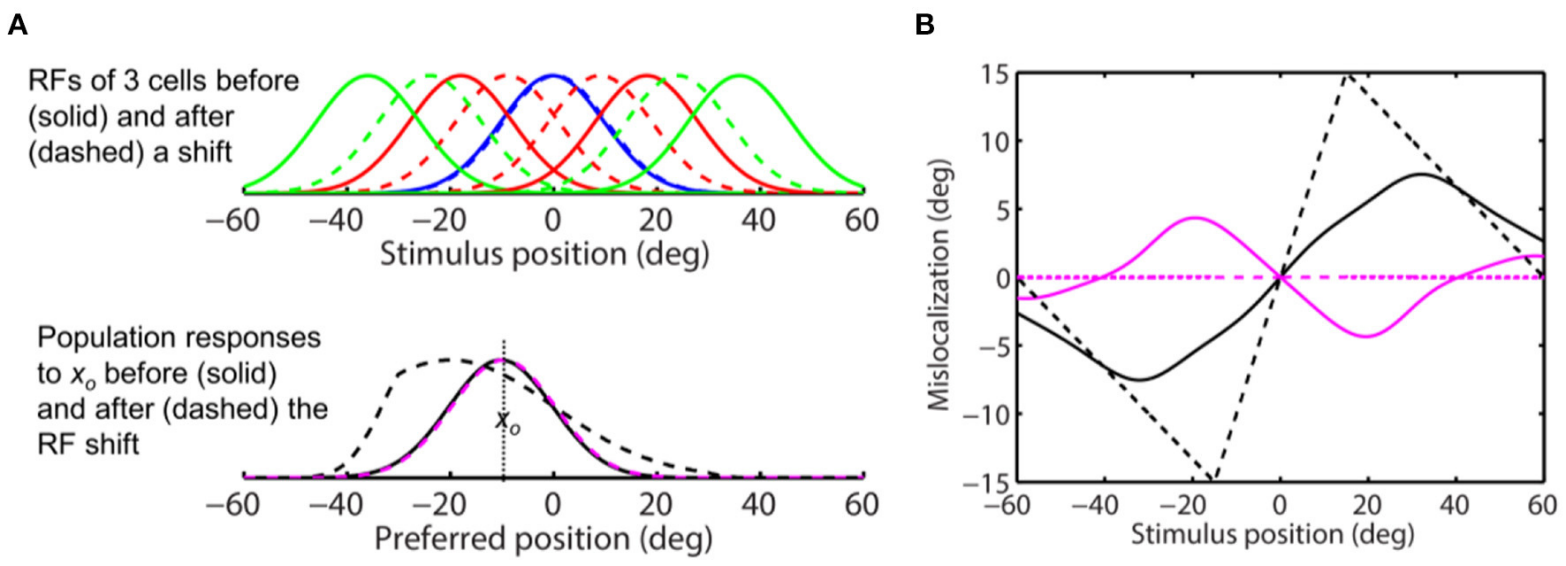
Perceptual consequence of convergent RF shifts. The target is at 0 deg. **(A)** (Top) RFs of five arbitrary cells before (solid) and after (dashed) the convergence. The “blue” cell tune to the target (0 deg) does not shift. (Bottom) the population responses of all cells to stimulus position *x*_*o*_ = −10 deg before (solid) and after (dashed) the RF convergence. The dashed black and magenta curves are the post-convergence responses plotted against the pre- and post-convergence preferred positions, for the unaware and aware decoders, respectively. **(B)** Perceptual mislocalization as a function of stimulus position relative to the target (0 deg). The results depend on whether the decoder is aware (magenta) or unaware (black) of the RF convergence, and whether the decoder uses the peak (dashed) or center-of-mass (solid) of population responses. The aware center-of-mass decoder (solid magenta) considers the change of the cells' covering density (see text); it predicts convergent mislocalization as stimuli to the left and right of the target (over a range of about 40 deg) have positive and negative mislocalization, respectively. The aware peak decoder (dashed magenta) predicts no mislocalization. The unaware center-of-mass and peak decoders (solid and dashed black) both predict divergent mislocalization.

Figure 8A, top panel, shows RFs of a few cells before (solid) and after (dashed) converging toward the target at 0 deg. Each RF is a Gaussian with σ = 10 deg. The “blue” cell tuned to the target location has no shift. The bottom panel shows the population responses to a stimulus at −10 deg before (solid) and after (dashed) the RF convergence. The dashed magenta and dashed black curves are population responses for aware and unaware decoders, respectively, showing no shift and a divergent shift (away from the target), respectively. We use both the center-of-mass and peak decoders to determine perceptual mislocalization for both the aware (Figure 8A, dashed magenta) and unaware (Figure 8A, dashed black) population responses, relative to the pre-shift baseline (Figure 8A, solid black). The results are shown in Figure 8B. As expected, the center-of-mass aware decoder (solid magenta) predicts convergent mislocalization: stimuli to the left and right of the target (over a range of about 40 deg) have positive and negative mislocalization, respectively. The predicted convergent mislocalization will be even stronger in two-dimensional space because the change of cells' covering density will be greater. The peak aware decoder (dashed magenta) predicts no mislocalization. The center-of-mass and peak unaware decoders (solid and dashed black) both predict divergent mislocalization. The maximum 15 deg divergent mislocalization at 15 deg distance predicted by the peak unaware decoder (dashed black in Figure 8B) can be understood: stimuli at this distance activate cells originally tuned to 30 deg distance but converged toward the target by 15 deg. Thus the unaware decoder mistakes stimuli at 15 deg as stimuli at 30 deg.

We now consider yet another new factor: attention at the target may not only produce convergent RF shifts but also modulate neuronal response strength. In fact, in a period before saccades, both LIP and FEF neurons tuned near the target location have enhanced visual responses while those tuned to locations further away from the target have suppressed visual responses (Schall et al., 1995; Falkner et al., 2010). In the above, we only considered the effect of RF shifts on population responses. We need to include the effect of the response modulation as well.

Based on the experimental data (Schall et al., 1995; Falkner et al., 2010), we consider an attentional modulation factor *g*(|*x*_*c*_|) as a function of the cRF-to-target distance |*x*_*c*_| (Figure 7D). To combine the effects of both the convergent RF shift and response modulation, we now replace the response function *f* (*x*_*p*_ – *x*_*s*_) by the product:


(10) $$f(x_{p}\;-\;[x_{s}\;-\;c(|x_{c}|)\;x_{c}])g(|x_{c}|)$$

The *f* (.) part is the same as before (Eq. 9). The attentional modulation factor *g*(|*x*_*c*_|) is peaked at the target |*x*_*c*_| = 0, and is greater and less than 1, respectively, for small and large |*x*_*c*_|, and stays at 1 (no modulation) for very large |*x*_*c*_|. Since *g*(|*x*_*c*_|) is not a function of stimulus position *x*_*s*_, the RFs as a function of *x*_*s*_ will just be scaled by *g*(|*x*_*c*_|) without changing their shapes (including preferred positions). On the other hand, *g*(|*x*_*c*_|) will change the shapes of the population responses as a function of *x*_*p*_, because |*x*_*c*_| depends on *x*_*p*_ (Eq. 8). In particular, for a stimulus close to and away from the target, its population response will be “pulled” toward and “pushed” away from the target by *g*(|*x*_*c*_|), respectively, compared with the no-modulation case, generating convergent and divergent mislocalization, respectively. Since the center excitation of attention is stronger than surround inhibition (Figure 7D), the main effect is convergent mislocalization for stimuli near the target.

Thus for stimuli close to the target, attentional modulation introduces a convergent component to population responses, increasing the convergent (compressive) mislocalization predicted by aware decoders. If the modulation is extremely strong, it may even make population responses of *unaware* decoders converge toward the target.

**Figure 9:**
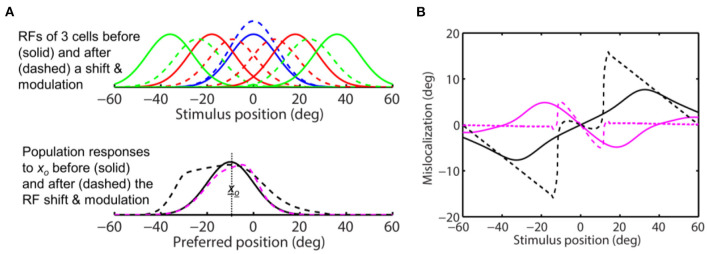
Perceptual consequence of convergent RF shifts and response modulation. The target is at 0 deg. The maximum response enhancement (at the target) is 25%. The format is identical to that of Figure 8. For stimuli close to the target, the aware center-of-mass and peak decoders (solid and dashed magenta) and the unaware peak decoder (dashed black) all predict convergent mislocalization. The unaware center-of-mass decoder predicts divergent mislocalization. The unaware peak decoder also predicts divergent mislocalization for stimuli away from the target.

To get a better sense of all the effects together, we ran simulations using a difference of Gaussians as the modulation factor:


(11) $$\begin{array}{l} g(|x_{c}|)\;=\;1\;+\;s\;(\;\exp[\;-\;|x_{c}{|}^{2}/(2\sigma_{e}^{2})]\;\\ \;-\;b\;\exp[\;-\;|x_{c}{|}^{2}/(2\sigma_{i}^{2})]) \end{array}$$

where σ_*e*_, σ_*i*_, and *b* determine the shape of the function, and *s* scales the function to determine the modulation strength. In Figure 7D, we let σ_*e*_ = 10 deg, σ_*i*_ = 25 deg, and *b* = 0.5 to produce a *g*(|*x*_*c*_|) similar in shape to the measured one in LIP (Falkner et al., 2010), and *s* = 0.5 so that the maximum attentional enhancement of responses (at the target) is 25% (Bushnell et al., 1981; Goldberg and Bushnell, 1981; Maunsell, 2015). We ran simulations with this modulation factor and the same convergent RF shift pattern as in Figure 8. The results are shown in Figure 9. As expected, the response modulation generates a convergent shift of the aware population response (Figure 9A, dashed magenta) and reduces the divergent shift of the unaware population response (dashed black) although the effects are relatively small. We again applied the center-of-mass and peak decoders to calculate perceptual mislocalization as a function of stimulus position (Figure 9B). Now for stimuli close to the target, both the center-of-mass and peak aware decoders (solid and dashed magenta), and the peak unaware decoder (dashed black), predict convergent mislocalization. The center-of-mass unaware decoder (solid black) still predicts divergent mislocalization. For stimuli further away from the target, the peak unaware decoder (dashed black) also predict divergent mislocalization.

**Figure 10:**
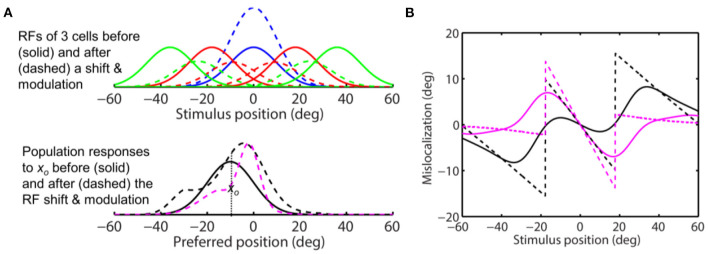
Perceptual consequence of convergent RF shifts and response modulation. The target is at 0 deg. The maximum response enhancement (at the target) is 100%. The format is identical to that of Figures 8, 9. For stimuli near the target, all the four decoders predict convergent mislocalization. Unaware decoders (solid and dashed black) still predict divergent mislocalization for stimuli away from the target.

We then repeated the simulation with *s* = 2 in Eq. 11 so that the peak attentional enhancement of responses (at the target) is 100%. The results in Figure 10A show sizeable convergent shifts for both the aware and unaware population responses (bottom panel, dashed magenta and black). With such large response modulation, all four decoders predict convergent mislocalization for stimuli close to the target (Figure 10B). This is consistent with a previous model (Hamker et al., 2008) which appeared to use an even larger response increase at the target location (300% in the first layer of the model) to generate convergent mislocalization. However, unaware decoders (solid and dashed black) still predict divergent mislocalization for stimuli away from the target.

We conclude that convergent RF shifts may predict convergent, divergent, or no mislocalization, depending on multiple factors including whether or not decoders are aware of the RF shift, whether or not aware decoders take cells' covering density into account, the strength of attentional modulation, and the stimulus-to-target distance. The same-direction hypothesis is correct for aware decoders that consider cells' covering density. It is also likely to be correct for aware decoders when stimuli are near the target and attentional modulation is present. Unaware decoders tend to predict a divergent mislocalization unless attentional modulation is extremely strong and stimuli are near the target.

## Discussions

In this paper, we analyzed differences between tuning curves and the corresponding population responses, and applied the analysis to determine how population responses change with two types of spatial tuning-curve shifts, namely the forward and convergent RF remapping (Duhamel et al., 1992; Umeno and Goldberg, 1997; Kusunoki and Goldberg, 2003; Zirnsak et al., 2014; Wang et al., 2016; Yang et al., 2019). We found that in the absence of response modulation, forward/convergent RF shifts alone produce either no shift or backward/divergent shifts of the population responses, depending on whether or not decoders are aware of the RF shifts. Since forward RF shifts, whether the forward jump or expansion, are assumed to be a uniform translation which does not change the density of cells covering different locations, the perceptual consequence simply follows the population responses: there should be no mislocalization and backward mislocalization for the aware and unaware decoders, respectively. This conclusion holds even when the increasing RF size with eccentricity, which breaks translational invariance, is considered.

The perceptual consequence of convergent RF shifts, however, is more complicated. For unaware decoders, the perception also simply follows the population response which predicts a divergent mislocalization. In contrast, for aware decoders, convergent RF shifts increase the density of cells covering the target area. Even though convergent RF shifts do not change the shape of the population response for aware decoders, perceptual decoding of the population response depends on whether a specific decoder takes into account the change of the cells' covering density (density-sensitive) or not (density-insensitive). As examples, we considered the center-of-mass aware decoder and the peak aware decoder. The former is density sensitive and predicts a convergent mislocalization whereas the latter is density insensitive and predicts no mislocalization. Thus, without response modulation, the same-direction assumption is correct for convergent RF shifts *only* when aware and density-sensitive decoders are used (Figure 8B, solid magenta). In all other cases, the same-direction assumption is false.

We then included the effect of attentional modulation which enhances responses at and near the saccade target (the attentional locus), and suppresses responses away from the target (Schall et al., 1995; Falkner et al., 2010). The effect of this modulation *alone* is straightforward: it produces convergent and divergent mislocalization for stimuli near and away from the target, respectively. When attentional modulation and RF shifts are combined, they predict a variety of mislocalization patterns, as demonstrated by our simulations, which depend on the parameters of the response modulation and RF shifts (including the strengths and ranges), decoders' awareness of the RF shifts, aware decoders' sensitivity to cells' covering density, and the stimulus-to-target distance. Using physiologically plausible parameters for convergent RF shifts and response modulation in Figures 7B, C, we found that aware and unaware decoders predict mostly convergent and divergent mislocalization, respectively (Figure 9B). Not surprisingly, when the attentional modulation is extremely strong, all decoders predict convergent mislocalization for stimuli near the target but unaware decoders still predict divergent mislocalization for stimuli away from the target (Figure 10B). Most studies in the literature seem to assume (often implicitly) unaware decoders. Since for unaware decoders convergent RF shifts do not change cells' covering density (Figure 7C), convergent mislocalization can occur only through strong attentional response modulation and only for stimuli near the attentional locus.

Although we only simulated the combined effect of attentional modulation and convergent RF shifts, it is easy to image the combined effect of attentional modulation and forward RF shifts. The forward RF shifts alone generate either no mislocalization (aware decoders) or backward mislocalization (unaware decoders). Attentional modulation, through its center excitation and surround inhibition, will add a convergent and divergent mislocalization component for stimuli close to and away from the target (or any attentional locus), respectively.

We now briefly summarize psychophysical data of perisaccadic perceptual mislocalization, which has been interpreted as reflecting imperfections of the mechanisms for transsaccadic visual stability (TSVS) (Matin and Pearce, 1965; Honda, 1991), and after the discovery of perisaccadic RF shifts (a specific mechanism for TSVS), as a perceptual consequence of the RF shifts (Ross et al., 2001; Kaiser and Lappe, 2004; Zirnsak et al., 2014). In a typical experiment, a probe stimulus (e.g., a dot or line) is flashed at various times around saccade onset, and subjects report the remembered stimulus location after the saccade. Over a window of about 150 ms around saccade onset, the probe is mislocalized with a translational component along the saccade axis and a compressive component toward the saccade target (Honda, 1991; Ross et al., 1997). The translational and compressive components are stronger, respectively, in the absence and presence of a post-saccadic visual reference, such as a ruler (Lappe et al., 2000). The translational mislocalization is in the saccade direction (forward) around the saccade onset, and disappears, or reverses the direction (backward), around the saccade offset (Honda, 1991; Lappe et al., 2000; Schlag and Schlag-Rey, 2002).

As we mentioned in the Introduction, the commonly held same-direction assumption posits that the forward and convergent RF shifts are responsible for the forward (translational) and convergent (compressive) perceptual mislocalization, respectively. Our analysis and simulations, however, cast some doubts on this assumption. First, the forward RF shifts predict either no mislocalization (aware decoders) or backward mislocalization (unaware decoders). Since forward RF shifts occur around the saccade onset, they cannot explain the observed forward mislocalization in that period. An exception to the forward mislocalization around the saccade onset is the study of Jeffries et al. (2007) who found backward mislocalization in monkeys. This is consistent with unaware decoders' prediction. However, that study differed from others in one aspect (in addition to monkey vs. human subjects): visual feedback of the veridical stimulus position was provided at the end of each trial. Further studies are needed to sort out the impact, if any, of this difference. Second, with reasonable parameters and for stimuli near the target, convergent RF shifts and attentional modulation together predict convergent mislocalization *only* for aware decoders (Convergent RF shifts *alone* predict convergent mislocalization only for aware *and* density-sensitive decoders.). Unaware decoders predict little or divergent mislocalization. Even with 100% attentional enhancement at the target, unaware decoders still predict divergent mislocalization for stimuli further away from the target. Without independent information on the brain's choice of aware vs. unaware decoders, we cannot determine whether or not convergent RF shifts explain convergent mislocalization.

There are other reasons to doubt the same-direction assumption. Convergent mislocalization depends on a post-saccadic visual reference such as a ruler or any visible background (Lappe et al., 2000). It is unknown whether or not convergent RF shifts depend on such reference but since convergent shifts in FEF and LIP appear around saccade onset or even in the delay period well before saccades (Zirnsak et al., 2014; Yang et al., 2019), they are unlikely to be dependent on post-saccadic references. A study showed that perceptual compression can occur without a post-saccadic reference if the stimuli are weak with near threshold luminance and observers dark adapt (Georg et al., 2008). However, convergent RF shifts are measured with supra-threshold stimuli (as weak stimuli would evoke too few spikes to measure the shifts efficiently). Moreover, forward remapping has been measured with both briefly flashed stimuli (Wang et al., 2016) and stimuli persisting to the end of trials (Duhamel et al., 1992). In contrast, mislocalization measurements appear to require brief stimuli (more on this later). Additionally, it is unclear how forward RF shifts may explain both the forward mislocalization at saccade onset *and* the backward mislocalization at saccade offset. Finally, perisaccadic RF remapping is regarded as a physiological mechanism for TSVS. Perisaccadic mislocalization, in contrast, is visual distortion or instability. The same-direction assumption has the conceptual difficulty of asserting that the stability mechanism directly causes instability. It would be more reasonable to assert that imperfect aspects of RF remapping generates residual instability. For example, forward RF remapping may start too early, before the saccade onset (Duhamel et al., 1992). However, this would simply predict an early start of either backward mislocalization (unaware decoders) or no mislocalization (aware decoders), still contradicting the same-direction assumption and the observed forward mislocalization before the saccade onset.

If the same-direction assumption is at least questionable, what then could be responsible for transsaccadic perceptual mislocalization? Traditional models assume that translational mislocalization results from the brain's poor estimate of eye position used to compensate for saccade-induced retinal shifts of stimuli (Matin and Pearce, 1965; Honda, 1991). Specifically, a slow-changing estimate that first leads but then lags the actual eye position during a saccade explains the forward and backward mislocalization around the saccade onset and offset, respectively. Pola (2004) argues that when latency and persistence of visual responses to flashed stimuli are considered, a delayed but otherwise veridical eye-position estimate can account for the translational mislocalization. Interestingly, Teichert et al. (2010) show that when temporal characteristics of visual responses to different stimuli are considered, the eye-position estimate that eliminates mislocalization for *persistent* stimuli produces the observed translational mislocalization for *flashed* stimuli. However, these models do not explain convergent mislocalization. Note that these models focus on eye-position estimates whereas our study focuses on RF remapping. Eye-position estimates rely on extraretinal signals but may also be influenced by retinal stimuli (Teichert et al., 2010). Conversely, RFs process retinal inputs but their remapping depends on extraretinal signals such as CD (Sommer and Wurtz, 2006). So the two approaches are not completely independent and may be combined in future research. Also note that temporal characteristics of visual responses to stimuli (such as latency and persistence) do not change our analysis on the perceptual consequences of RF remapping as long as the stimuli are timed to produce the remapping (such as the perisaccadic stimuli we consider). Similarly, early or late decoding does not change our results as long as the decoders act on remapped RFs. Clearly, our results are irrelevant if a stimulus does not produce RF remapping or perceptual decoders act on RFs at a time without remapping.

Cicchini et al. (2013) found that a perisaccadically presented bar is attracted to another bar presented either pre- or post-saccadically. The interaction occurs over an oriented region of spatial and temporal separations between the bars, characteristic of motion detectors (Adelson and Bergen, 1985). Interestingly, the forward RF expansion can also be interpreted as a spatiotemporal orientation because around saccade onset, stimuli closer to a cell's cRF and fRF evoke visual responses with shorter and longer latencies, respectively (Wang et al., 2016). Thus, forward expanded RFs may be viewed as CD-enabled high-speed motion detectors that measure spatiotemporal correlation in retinal image motion across saccades, not for perceiving the motion, but for linking pre- and post-saccadic retinal images to achieve TSVS. Perisaccadic mislocalization could then occur if this motion detection is imperfect (Cicchini et al., 2013). However, this possibility again cannot explain convergent mislocalization because saccade-induced retinal motion is largely uniform across the retina at a given time.

Surprisingly, patterns similar to perisaccadic mislocalization, with both the translational and convergent components, have been produced by simulating saccade-like retinal motion without the actual saccade (Ostendorf et al., 2006; Shim and Cavanagh, 2006). Ostendorf et al. argue that earlier experiments that failed to find convergent mislocalization with simulated motion either did not match simulated motion and saccade-induced motion, or were not designed to measure compression. Just like perisaccadic mislocalization which starts before the saccade onset, the motion induced mislocalization starts before the motion onset. Such motion induced mislocalization of flashed stimuli in the absence of eye movements is known as the flash-lag effect (Brenner et al., 2006; Watanabe and Yokoi, 2006). Although the mechanism of the flash-lag effect itself is still debated (Nijhawan, 2002; Khoei et al., 2017), it likely involves uncertainty and delays in processing brief stimuli whose noisy memory representations interact with other visual references. Indeed, most demonstrations of both the flash-lag and perisaccadic mislocalization effects use flashes of less than 10 ms. The flash-lag effect greatly decreases or largely disappears for flash durations longer than 100 ms (Lappe and Krekelberg, 1998; Cantor and Schor, 2007). This makes sense because longer stimuli produce more reliable neural representations which are less vulnerable in memory. Similarly, although perisaccadic mislocalization of brief stimuli can be many degrees of visual angle, we never notice it in our daily life suggesting that it may not exist for persistent stimuli (Teichert et al., 2010). [Alternatively, one could argue that saccades suppress persistent objects in daily life much more strongly than brief stimuli in mislocalization studies. This, however, contradicts the fact that saccadic suppression is stronger for magnocellular stimuli such as flashes (Ross et al., 2001)]. Thus, perisaccadic mislocalization might be a version of the flash-lag effect (Teichert et al., 2010) unrelated to saccades per se or mechanisms of TSVS.

Since saccades generate retinal motion which then produces mislocalization by itself, how can we determine perceptual consequences of RF shifts without the confound of the saccade-induced retinal motion? Total darkness would eliminate retinal motion but it is hard to achieve when initial fixation points, targets, and probes have to be visible. Fortunately, convergent RF shifts can be generated by attention in time periods well separated from saccades (Neupane et al., 2016; Yang et al., 2019). One can thus measure attention induced mislocalization without saccades and the associated retinal motion. Suzuki and Cavanagh (1997) did exactly such a study. They used a Vernier task to measure attentional mislocalization, and found that probe stimuli were repelled away from, not attracted toward, the attentional locus. This result contradicts the same-direction assumption, and is consistent with unaware decoders' prediction of divergent mislocalization (Figures 8B, 9B, black curves). Indeed, they discussed a model which uses unaware decoders without attentional modulation. However, the maximum mislocalization they measured was only about 0.3 deg, much smaller than typical perisaccadic mislocalization at similar stimuli-to-target distance. Perhaps the model could accommodate the small mislocalization by using the fact that convergent RF remapping is relatively weak (Neupane et al., 2016) and only a small fraction of cells show convergent RF shifts (Yang et al., 2019). Another issue is that since they measured mislocalization by comparing two flashed Vernier lines, the result must be the difference between the two lines' mislocalizations (which may also contribute to the small effect). To explain the data, the model has to assume that attention repels the near line more than the far line, or attract the near line less than the far line, in the experiment. This is possible (Figure 9B) but requires independent verification.

In conclusion, our work suggests that there is no strong theoretical support for the commonly-held same-direction assumption, which links perisaccadic forward and convergent RF shifts to perisaccadic forward (translational) and convergent (compressive) perceptual mislocalization. Perisaccadic mislocalization might be a version of the flash-lag effect caused by saccade-induced retinal motion instead of by saccades per se or by mechanisms of TSVS. However, although forward RF shifts cannot explain forward mislocalization, convergent RF shifts, together with attentional response modulation, may contribute to convergent mislocalization particularly for aware decoders. To resolve this issue, one has to address the key open question of whether or not perceptual decoders in the brain are aware of the RF shifts.

## Data availability statement

The original contributions presented in the study are included in the article, further inquiries can be directed to the corresponding author/s.

## Author contributions

NQ, MZ, and MG conceived and discussed the project and edited the manuscript. NQ did the simulations and wrote the first draft of the manuscript. All authors contributed to the article and approved the submitted version.
